# Abiotic Stress Tolerance in Crop and Medicinal Plants

**DOI:** 10.3390/plants12244167

**Published:** 2023-12-15

**Authors:** Anelia G. Dobrikova

**Affiliations:** Institute of Biophysics and Biomedical Engineering, Bulgarian Academy of Sciences, Acad. G. Bonchev Str., Bl. 21, 1113 Sofia, Bulgaria; aneli@bio21.bas.bg

## 1. Introduction

Climate change and the increased need for crop production highlight the urgent importance of introducing crops with increased tolerance to adverse environmental conditions [1]. Many studies have focused on creating and studying various crop species (genotypes, varieties, cultivars, hybrids, etc.) resistant to different abiotic stress factors, especially drought, salinity, light, extreme temperatures, heavy metals, etc., applied alone or in combination. Breeding and genetic modification methods intended for crop improvement have created many plant species with greater resistance to abiotic stress [2]. The non-genetic approach to enhancing crop yields in stressful environments involves the use of exogenous phyto- and biostimulants [3], as well as primary and secondary plant metabolites [4,5]. Since the effectiveness of these strategies in improving plant stress tolerance has been proven, they have the potential for widespread application in the future. In addition to these strategies, a lot of attention has been paid to protecting plants’ photosynthetic function under abiotic stress [6,7]. There is evidence to suggest that the use of strategies to improve the photosynthetic performance under stress conditions can increase plant yields, which has led to a growing interest in studying photosynthetic tolerance as a tool to enhance plant production under adverse environmental conditions [6]. Moreover, environmental stress has a strong impact on the photosynthetic membranes of plants, causing damage on multiple levels by affecting the ultrastructure of thylakoid membranes, pigment content, protein and lipid compositions [7]. This fact emphasizes the importance of studying the adaptation mechanisms of photosynthetic apparatus to achieve a deeper understanding of plant stress responses, which will be useful in the actual selection of crop-tolerant genotypes. 

This Special Issue, “Abiotic Stress Tolerance in Crop and Medical Plants” (Volume I and II), collects papers on new approaches to the development of strategies to increase the abiotic stress tolerance of crop and medicinal plants. It also focuses on studying the photosynthetic adaptation mechanisms in strategic crops and medicinal plants to changing environmental conditions for the fast detection and screening of their stress tolerance in the context of climate change. The papers published in the present Special Issue (consisting of 27 original articles and 2 reviews) address various environmental stress factors such as drought, salinity, light stress, cold stress, heavy metal toxicity, etc., applied individual or in combination. They provide important insights into the underlying mechanisms of plant tolerance, as well as practical ways to alleviate the harmful effects of environmental stress by different means such as plant metabolites, signaling molecules, phytoprotectants, biostimulants, etc. Some papers also demonstrate the adaptation of different plant genotypes to individual or combined stress factors. The insights provided by all of these studies will help us to better understand the tolerance mechanisms of plants against various abiotic stress factors, helping to ensure future food security.

## 2. Tolerance Mechanisms in Crop Plants

Unfavorable environmental changes affect the biochemical and physiological processes, growth, and development of crop plants and thus can significantly reduce crop yield and quality. Crop plants have developed a wide set of responses to tolerate environmental stress depending on their capacity for adaptation [1,5]. In this Special Issue, several articles explore the morphological, biochemical, and physiological responses of important crop plants (or different genotypes) and their adaptation to environmental stress, as well as the different ways to increase their resistance to drought stress (contributions 1–5), osmotic and salt stress (contributions 6–12), and the combined effects of drought and salinity (contributions 13–15).

Information about the application of exogenous biostimulants and phytohormones for improving crop stress tolerance is also included in this Special Issue. Rady et al. (contribution 1) propose the use of exogenous gibberellic acid and diluted bee honey as biostimulants to ameliorate the drought tolerance of bean plants, and in their study, they achieved improved growth and productivity under water-deficient conditions. Al Kahtani et al. (contribution 6) demonstrate the possible effectiveness of applying *Bacillus thuringiensis* and silicon to endow lettuce plants with tolerance to salinity. Stassinos et al. (contribution 7) suggest that seed priming with spermidine influences the responses to salt stress of three rapeseed cultivars and demonstrate an improvement in their tolerance to high-saline conditions. Another study by Stefanov et al. (contribution 12) discusses the protective effects of sodium nitroprusside on the photosynthetic function of sorghum plants subjected to salt stress. Kunene et al. (contribution 4) show that a drought-tolerant Bambara groundnut genotype can be recognized during the early growth stage by screening for drought-tolerance markers, and this knowledge can be used for improving crop production. Yue et al. (contribution 15) propose that *OsmiR535* has the potential to be a target for the genetic editing of plants’ drought and salt tolerance which can be used as a new marker for molecular breeding in rice plants.

Elkelish et al. (contribution 16) report that the exogenously applying ascorbic acid enhances the cold stress tolerance of tomato plants. Popova et al. (contribution 17) reveal that alternative electron pathways are involved in the photosynthetic responses to high-light intensity and low temperature by studying the acclimation of two *Arabidopsis thaliana* species (wild-type and mutant *lut2*) to both stress factors.

Other articles published in this Special Issue deal with the mitigation of heavy metal stress, showing that the application of trehalose alleviates cadmium toxicity in mung bean plants by enhancing the photosynthetic activity and antioxidant defense system (contribution 18), and 5-aminolevulinic acid increases lead tolerance in sage plants (contribution 19). Zishiri et al. (contribution 20) identified several maize genotypes (inbred lines) with genetic variations conducive to aluminum tolerance and explains that they could be used by breeders in maize breeding programs to reduce yield losses.

The review paper by Giraldo Acosta et al. (contribution 21) proposes the application of melatonin as a natural safener in herbicide treatments of crop plants, highlighting its excellent capabilities to reduce herbicide damage and activate antioxidant defense. Melatonin has been described as a hormonal molecule that can stimulate the functions of plants under various abiotic and biotic stresses.

## 3. Tolerance Mechanisms in Medicinal Plants

Abiotic stress factors such as drought, salinity, high light, extreme temperatures, etc., can also reduce the quality and productivity of medicinal plants by disrupting their biochemical, metabolic, and physiological processes [8,9]. It has been also established that the application of various biostimulants like phytohormones, plant-growth-promoting Rhizobium, nanomaterials, and biochar can improve the resistance of medicinal plants to stress by stimulating the biosynthesis of primary and secondary metabolites and phytohormones and increasing their chlorophyll contents, antioxidant potential, and nutrient uptake, thereby reducing oxidative stress [9].

This Special Issue also includes studies on the tolerance mechanisms of medicinal plants, as well as different treatments that can reduce the harmful effects of abiotic stresses to achieve the high-quality production of medicinal and aromatic plants under environmental stress. The review by Hlongwane et al. (contribution 22) highlights the effectiveness of plant-growth-promoting rhizobacteria in alleviating the harmful effects of abiotic stress factors such as salt and drought in the medicinal plant *Lessertia frutescens*, whose curative ability is related to its enriched phytochemical composition, which includes amino acids, flavonoids, and triterpenoids. The study by Sichanova et al. (contribution 23) evaluates the influence of different concentrations of two types of nanofibers (derivatives of aspartic acid with silver ions) on the growth parameters, antioxidant activity, and steviol glycoside content of micropropagated *Stevia* plants. The authors of this study suggest that the application of silver salt nanofibers appears to be an effective strategy for enhancing the presence of metabolites relevant to human health and addressing various abiotic and biotic stresses.

Szekely-Varga et al. (contribution 24) establish the stress responses and the relative tolerance of two commercial lavender varieties to drought and salinity, showing the relevant mechanisms involved in their tolerance. They also describe the possibility of using biochemical stress biomarkers for the quick screening and selection of lavender genotypes better adapted to climate change scenarios.

El-Sherbeny et al. (contribution 25) discuss the morphoanatomical features and biochemical responses (such as an increase in the contents of phenols, flavonoids, alkaloids, and tannins, and increased antioxidant activity) of two medicinal vascular plants species—*Artemisia monosperma* and *Limbarda crithmoides*—developing in the arid coastal habitats of Egypt. The authors describe the adaptation mechanisms used by these plant species and provide insights into the defense and survival strategy of these plant species under extremely harsh conditions.

Zhao et al. (contribution 26) indicate that light intensity has a regulatory role in the increasing accumulation of flavonoids, which allows the alpine herbal plant *Sinopodophyllum hexandrum* to adapt to the elevated altitude associated with high-light intensity. It has been also found that higher light intensities are correlated with greater flavonol, flavonoid, and anthocyanin contents as well as with higher anthocyanin/total flavonoid and anthocyanin/total flavonol ratios.

In another study, the tolerance mechanisms of the medicinal and aromatic plant clary sage (*Salvia sclarea*) against excess zinc (Zn) stress were evaluated by studying observed changes in leaf pigment and phenolic content, photosynthetic performance, nutrient uptake, and the characteristics of the leaf structure (contribution 27). This study reveals that clary sage is an appropriate plant for the phytoextraction of Zn from polluted soils, as well as for the phytoremediation of heavy-metal-contaminated soils. In addition, El-Shora et al. (contribution 19) suggest that antioxidant defense mechanisms can improve the heavy metal tolerance of sage plants (*Salvia officinalis*) and recommend the application of 5-aminolevulinic acid to alleviate lead stress.

## 4. Conclusions

The present Special Issue provides useful insights into the complex interactions between plants and the changing environment, shedding light on the different strategies that crop and medicinal plants use to adapt to and mitigate the harmful effects of abiotic stresses, which would have crucial effects on sustainable food and pharmaceutical production. This Special Issue also presents studies of new tolerant crop genotypes and different eco-friendly ways to improve the tolerance of plants under unfavorable environmental conditions. The effectiveness of different phytoprotectants and/or biostimulants in inducing effective tolerance mechanisms in plants against environmental stress is also discussed. The sharing of such valuable insights must continue to help develop a sustainable future agriculture that is better adapted to environmental changes and environmental pollution.

I express my deepest gratitude to all authors who accepted the opportunity to present their research in this Special Issue and thank them for their efforts in studying abiotic stress tolerance in plants.
